# Clinicopathological study of male breast carcinoma: 24 years of experience

**DOI:** 10.4103/0256-4947.55314

**Published:** 2009

**Authors:** Parveen Shah, Irfan Robbani, Omar Shah

**Affiliations:** a Department of Pathology, Sher-i-Kashmir Institute of Medical Sciences, Srinagar, Jammu and Kasmir, India; b Department of Radiodiagnosis, Sher-i-Kashmir Institute of Medical Sciences, Srinagar, Jammu and Kasmir, India; c Department of Surgery, Sher-i-Kashmir Institute of Medical Sciences, Srinagar, Jammu and Kasmir, India

## Abstract

**BACKGROUND AND OBJECTIVES:**

Because breast cancer in men is rare, few patients are available for prospective studies. To learn more about its epidemiology, risk factors, clinical features, genetics and pathology in our country, we conducted a retrospective study of all cases seen in recent decades at our institution.

**PATIENTS AND METHODS:**

We identified each case of male breast cancer in the database at the Sher-i-Kashmir Institute of Medical Sciences, Srinagar, Kashmir, India, between the years 1983 to 2007.

**RESULTS:**

We identified only 32 cases of male breast cancer over the 24-year period. Male breast cancer accounted for 32 (2.8%) of 1141 resected breast specimens, which included all breast lesions and 32 (4.1%) of 780 breast cancer cases. Of the 32 cases, 20 (62.5%) had various associated risk factors. Invasive ductal carcinoma was seen in 30 cases (93.7%). Of 20 cases that underwent molecular studies, 16 (80%) patients had estrogen receptor positivity whereas 14 (70%) had progesterone receptor positivity. Six cases (30%) overexpressed HER2 and p53. The *BRCA2* mutation was observed in 4 cases (40%) while no patient presented with the *BRCA1* mutation.

**CONCLUSION:**

An incidence of 4.1% for male breast cancer indicates that this disease is not as uncommon as presumed in this part of the world. Breast cancer in men seems more frequently to be hormone receptor positive and the *BRCA2* mutation confers a significant risk to men.

Breast cancer in males is relatively uncommon, accounting for less than 1% of all breast cancers and less than 1.5% of all malignancies in men.^1^–^3^ With the exception of Tanzania and countries of central Africa, male breast cancer is distributed uniformly throughout the world.^4^,^5^ The incidence of male breast cancer has remained stable over the past four decades. Because of this low incidence, unlike carcinoma of the breast in women, a limited number of patients are available for study. Even large oncological centers have relatively small numbers. It comes as a shock to many to learn that men too can get breast cancer.^6^ In the present study, we review our experience of 24 years with this disease, focusing on its epidemiology, risk factors, genetics, pathology and molecular markers.

## PATIENTS AND METHODS

For this retrospective study, we identified all male breast cancer patients seen from January 1983 to December 2007 at the Department of Pathology, Sher-i-Kashmir Institute of Medical Sciences, Srinagar, Kashmir. The history, including tumor size, histological and nuclear grades, was evaluated. The TNM classification system was used for tumor staging. Histological material for study was available in all cases. Data on epidemiology, risk factors, clinical assessment, genetics, pathology and molecular markers were the focus of study.

Immunohistochemical studies were done routinely in breast carcinoma cases seen in the last decade. In some cases reported earlier, immunohistochemistry was done as part of the present study. Molecular marker studies were done in 20 cases and genetic studies in 10 cases. For immunohistochemical studies, 5-μm sections of formalin-fixed, paraffin-embedded tissue were cut and placed on gelatin-coated slides. Estrogen receptor, progesterone receptor and p53 immunostaining were performed using the avidin-biotin complex peroxidase technique with the chromogem diaminobenzidine and with the microwave antigen retrieval technique. ER and PR nuclear stains were categorized as positive or negative. Positive staining was established as >50% of the cells showing distinctive nuclear stain; p53 immunoreactivity was interpreted using the criteria described by Thor and Yandell.^7^ The tumor was scored positive if cells showed strong uniform staining of the nuclei; as intermediate for weak or focal nuclear staining and as negative for no staining. ER, PR, p53 and HER2 protein, immunohistochemical studies were performed in 20 patients (62.5%) whereas 10 patients (31.2%) had *BRCA1* and *BRCA2* detection. Immunohistochemistry was the only method used for detection of *BRCA1* and *BRCA2* mutations. In 10 cases (31.2%) of male breast carcinoma, formalin-fixed, paraffin-embedded blocks were analyzed using monoclonal antibody for the *BRCA1* protein (anti-*BRCA1* [Ab-1], human [mouse]: epitope: 1-304 N-terminal aminoacids of BRCA1) and a polyclonal antibody for the *BRCA2* protein (anti-*BRCA2* [Ab-2], rabbit polyclonal Ig G: epitope: 3245-3418 of human *BRCA2*) (Oncogene Research Products/Calbiochem, Darmstadt, Germany) at a dilution of 1:300 and 1:100, respectively. Antigen retrieval was performed by heating the specimens in a pressure cooker for 6 minutes in citrate buffer (pH 6). The Envision system (Dako, Copenhagen, Denmark) was used for detection. Sections from positive breast invasive ductal carcinoma were used as positive controls, and negative controls were obtained by omitting the primary antibodies. The reaction was considered positive if more than 10% of the cells showed distinctive nuclear staining.^8^ HER2-protein expression was carried out by the immunohistochemistry method using monoclonal antibody (CB 11-NOVOC-Castra, Newcastle upon Tyne, UK) (TAB 250, Zymed, San Francisco CA, USA). The test was carried out on tumor tissue samples fixed in buffered formalin and embedded in paraffin wax. Based on an assessment of the intensity of the reaction product and percentage of positive cells, scoring of *HER2* expression was graded from 0 to 3+.^9^

## RESULTS

The incidence of carcinoma of the male breast was 2.8% (32/1141) of the total resected breast specimens, which included all breast lesions and 4.1% (32/780) of total breast cancer cases. Mean age at presentation was 55 years (range, 40 to 70 years), with the youngest patient being 40 years old and the oldest 70 years old. Of the 32 cases in this study, 20 cases (62.5%) had various risk factors for male breast carcinoma (Table 1). Testicular abnormalities with infertility were seen together in 3 cases (9.3%). One case had a history of radiation exposure and one had a positive family history and obesity. Sixteen (50%) patients had a painless breast mass (Table 2). Nipple and skin retraction was seen in 12 patients (37.5%). A retroaerolar presentation was the most common site. Twenty-two patients (69%) presented with nodal positivity. Twelve cases (37.5%) had 1-3 lymph nodes positive whereas 10 cases (31.2%) had more than 4 lymph nodes positive. In 10 cases (31.2%)—lymph nodes were free of tumor. Eighteen patients (56.2%) presented with stage III disease, but a significant number—12 (37.5%) and 10 (31.2%)-presented with stage II and stage I disease, respectively. No patient had stage IV carcinoma. Fine needle aspiration cytology smears and conventional pathologic features were evaluated. Cytology of aspirate smears revealed malignancy in 30 cases (93.7%). Material was inadequate in 2 cases (6.2%). Most of our cases (30 cases, 93.7%) were invasive ductal carcinoma, with one each (3.1%) of papillotubular carcinoma and adenoid cystic carcinoma (Table 3). In our series we saw no patient with Paget disease of the nipple. The various genetic and molecular markers are summarized in Table 4.

**Table 1:** Risk factors for male breast carcinoma.

Testicular abnormalities	
Undescended testis	3
Congenital inguinal hernia	Nil
Orchidectomy	Nil
Orchitis	2
Testicular injury	Nil
Infertility	3
Klinefelters syndrome	Nil
Positive family history	1
Obesity	4
Radiation exposure	1
Other factors	Nil
Breast cysts	4
Breast trauma	1
Gynecomastia	10

**Table 2:** Clinical features in 32 cases of carcinoma of the male breast.

Clinical Features	Invasive (n=28)	Non-invasive (n=4)
Mean age (years) (range)	55 (30-75)	56 (50-60)
Mean duration of symptoms (months) (range)	3 (1 month-1 year)	9 (6 months-1 year)
Mass	16/32 (50%)	4/32 (12.5%)
Mean size (cm) (range)	2 (0.5-4.5)	1.0 (1.0-2.0)
**Site**		
Retroaerolar	24/32 (75%)	4/32 (12.5%)
Upper outer quadrant	8/32 (25%)	-
Skin and nipple retraction	12/32 (37.5%)	-
Lymph node metastasis	22/28 (78%)	0

**Table 3:** Histopathological tumor type.

Invasive ductal carcinoma	30 (93.7%)
Papillotubular carcinoma	1 (3.1%)
Solid tubular	Nil
Schirrhous carcinoma	Nil
Lobular carcinoma	Nil
Adenoid cystic carcinoma	1 (3.1 %)
Paget's disease	Nil
**Total number of cases**	32

**Table 4:** Pathologic and genetic features of breast carcinoma in men.

Pathological Features	Total number of cases	Number of cases positive or immuno-reactive	
Estrogen receptor	20	16	80%
Progesterone receptor	20	14	70%
HER2	20	6	30%
p53 protein	20	6	30%
*BRCA1*	10	Nil	Nil
*BRCA2*	10	4	40%

## DISCUSSION

The phenotypic alterations of male breast tumor are not well studied, and experience is mainly inferred from that of female breast cancer. Although both diseases have similarities, there are notable differences in risk factors, prognosis and survival. Differences between male and female breast carcinoma have been noted; male breast carcinoma has a tendency to present at higher clinical stages and with more lymph node metastases. The incidence of breast cancer in males, unlike that in females, has not increased appreciably over the last 40 years.^3^ The incidence increases exponentially with age.^10^ In most western countries, men account for approximately 1% of cases of breast carcinoma. In Tanzania, by contrast, 6% of cases of breast cancer are diagnosed in men,^4^ and in countries of central Africa, a substantially higher proportion of cases of male breast cancer have been reported.^5^ The reasons for this geographic variability remain unclear. However, this may be due to a higher prevalence of liver disease (schistosomiasis or malnutrition), resulting in an increase in endogenous estrogens. In a study conducted at a tertiary care hospital in the eastern part of India, the reported incidence of male breast cancer was 0.6% among all male cancers and 2.5% among all breast cancer cases.^11^ In recently published data from a neighboring country (Pakistan), male breast cancer constituted 4.6% of all breast carcinomas.^12^ We report an incidence of 4.1% of the total breast cancer cases, which is higher than the generally reported incidence of 1% worldwide. This higher incidence is possibly due to our institute being a referral center. Many of the risk factors for breast cancer in men, as in women, may be hormonally driven. An elevated risk has been seen in patients with undescended testes, congenital inguinal hernia, orchidectomy, orchitis, testicular injury and infertility.^4^,^5^,^11^,^13^ In our study, there were 3 cases (9.3%) each of undescended testes and infertility. Orchitis was reported in 2 cases (6.2%). Other possible risk factors that relate to hormonal levels include obesity, which increases peripheral aromatization of estrogen, and cirrhosis, which results in a hyperestrogenic state.^14^,^15^ Estrogen has a profound effect on male breast tissue and is able to stimulate formation of acini and true lobules identical to those of the female breast. There have been many reports of breast tumors developing in men undergoing estrogen therapy for prostrate carcinoma that have proved to represent secondary involvement of an estrogen-altered breast as part of the metastatic process. Alterations of hormonal metabolism in men with breast cancer have been demonstrated, but no consistent pattern has emerged. Several studies have implied an association between testicular abnormalities and male breast cancer. Prior orchitis has been reported more frequently among men with breast cancer than among controls. Obesity, especially that occurring before the age of 30 years, may be another risk factor. The presumed mechanism is increased conversion of androgens to estrogens through peripheral aromatization. Four (12.5%) of our patients were obese and 10 (31.2%) patients had gynecomastia. A rare malignancy, male breast cancer has in epidemiological studies been associated with a prior history of gynecomastia.^16^,^17^ However, a recently conducted prospective study in men with gynecomastia confirmed an increased risk for testicular, skin and esophageal cancers whereas no prospective cases of male breast cancer were seen.^18^ It was concluded that before gynecomastia is included as a risk factor for male breast cancer, a man presenting with gynecomastia should be evaluated for the following: Is there a endogenous or exogenous hormonal cause? Has the patient been taking drugs that may cause gynecomastia? Is there a known accompanying disease causing a liver or gonadal injury? Can tumor disease in the testis, skin and breast be excluded on clinical grounds?

There are a few studies that have addressed meat consumption and the risk of male breast cancer.^19^,^20^ Conflicting reports have emerged, but in our study all 32 patients were red meat eaters. Although meat as a risk factor for getting male breast cancer has not been studied in a study from the same geographical region (Pakistan) showed an identical incidence of male breast carcinoma.^12^ It would be interesting to study meat consumption as an epidemiological risk factor, since most of the population in Kashmir and Pakistan consume red meat in large quantities.

Approximately 15% to 20% of male patients with breast cancer have a positive family history, although only 7% of the general male population has an affected family member.^21^,^22^ Therefore, researchers have suspected that some families may carry genetic mutations that provide an increased risk for breast cancer. In our series, one patient had a positive family history. This patient had a first-degree relative with breast cancer. In men, *BRCA1* does not appear to be associated with a significantly increased risk for breast cancer, although mutations in this gene have been described in affected men.^23^ However, men with *BRCA2* mutations are pre-disposed to breast cancer. This gene was first identified by Wooster who localized it to chromosome 13q12-13 and described multiple cases of breast cancer in men that showed linkage to this area.^24^ Families in which breast cancer has occurred and where at least one male has been affected have been reported to have a 60% to 76% chance of carrying *BRCA2* mutations.^25^ Thus, the presence of breast cancer in men within a family with documented breast cancer indicates a high likelihood of a *BRCA2* mutation. In 10 patients (31.2%) tested for genetic mutations *BRCA1* and *BRCA2, BRCA2* positively was seen in 4 cases (40%). No immunoreactivity was seen with *BRCA1.* One case (10%) of male breast carcinoma with a positive family history had *BRCA2* mutation positivity. Eleven percent to 40% of men with breast cancer carry this mutation.^26^ The highest known prevalence of *BRCA2* mutations in male patients with breast cancer is in Iceland, where a founder mutation accounts for 40% of all cases.^27^ Little is known about the distinguishing characteristics of breast cancer in men with *BRCA2* mutations, although men with a mutation may present with disease at an earlier age.^28^

Almost all of the histologic subtypes of breast carcinoma that have been diagnosed in women have also been reported in men. Approximately 90% of all breast tumors in men are invasive carcinomas, the remaining 10% are non-invasive. In our study, 30 cases (93.7%) were invasive carcinomas and the remaining were noninvasive. The proportion of noninvasive cancers is higher than that seen in women before the introduction of mammography and may be due to the small size of the male breast, which simplifies the detection of small breast masses.^29^ Almost all of the noninvasive cancers are ductal carcinomas in situ. Lobular carcinoma in situ is extremely rare because of the absence of terminal lobules in the normal male breast.^21^ Ductal carcinomas in situ of the male breast differ from that of the female breast in that 75% of cases are a papillary subtype and almost all cases are low to intermediate grade.^30^

In men, the predominant histologic subtypes of invasive carcinoma are infiltrating ductal carcinoma, which accounts for more than 80% of all tumors, and papillary carcinoma, which makes up about 5%.^31^ Lobular carcinoma is much less common in men than in women and represents only 1% of all cases.^21^ The rare subtypes, such as medullary, tubular, mucinous and squamous carcinomas, have all been reported in men,^31^ although they are slightly more uncommon than in women. Inflammatory carcinoma and Paget disease are seen with a similar frequency in men and women.^29^

Carcinomas of male breast have a higher rate of hormone receptor positivity than do carcinomas of the female breast when matched for tumor stage, grade and patient age.^32^ Our study indicates that 80% of male breast carcinomas are estrogen-receptor positive and 70% are progesterone receptor positive. This is in conformity with other studies.^21^,^33^

However, hormone expression up to 95% has been shown in a study by Wang-Rodriguez et al.^34^ Aberrant steroid receptor up-regulation as a consequence of constitutive activation of downstream targets in the estrogen-starved postmenopausal setting and in male breast patients have been postulated for hormone receptor expression.^35^

HER2, p53, bcl-2, cyclin D1 and epidermal growth factor (EGFR) are important in the pathogenesis and prognosis of breast cancer in women. In our data (Table 4), only in 20 cases (62.5%) were p53 and HER2 available. The *HER2* protooncogene encodes a membrane of the EGFR family, and is located on chromosome 17q21. This receptor is expressed in 20% to 30% of breast cancers in females and its positivity is related to high grade tumors, lymph node metastasis, a higher rate of disease recurrence, higher mortality and thus a poor prognosis. Further *HER2* status is a predictive factor of response to particular systemic therapies, notably trastuzumab. The College of American Pathologists (CAP) both consider *HER2* testing to be part of the standard workup for a newly diagnosed breast cancer.^36^,^37^

*P*53, a tumor suppressor gene, controls cell growth by including cell-cycle blockade, apoptosis and cell differentiation. A positive test for mutation indicates a poor prognosis. A review of literature revealed that between 18% and 58% of male breast cancers test positive for *P*53 by immunohistochemistry,^38^,^39^ which is confirmed by our results. Though carcinoma of male breast has a similarity to its female counterpart, it differs genetically. *BRCA1* and *BRCA2* can cause breast cancer in females, but only *BRCA2* mutation confers a significant risk to men. Of 32 cases of male breast carcinoma in our series, a genetic marker study was available in 10 cases (31.2%). Of these cases, 4 (40 %) cases were immunoreactive the *BRCA2* mutations and were also positive for estrogen and progesterone receptors. Among these four cases was one case with a documented family history of breast carcinoma. Higher rates of estrogen and progesterone receptor positivity may be observed in men than in women, but similar percentages may be expressed for *HER2* and p53. Larger studies for pathological markers will certainly reveal genetic abnormalities that play a role in the pathogenesis and also ascertain various prognostic markers. The cell surface glycoprotein E-cadherin (CDH1) is a key regulator of adhesive properties in epithelial cells. Germline mutations in CDH1 are well established as the defects underlying heredity diffuse gastric cancer (HDGC) syndrome along with an increased risk of lobular breast cancer (LBC) have been described in HGDC kindreds. Pleomorphic lobular carcinoma shares some characteristics with classic infiltrating lobular cancer, although it may also have distinctive features, thus raising the question of whether pleomorphic lobular carcinoma is a tumor of a lobular nature. In this context, several studies have demonstrated that the analysis of E-cadherin expression is a powerful tool for distinguishing lobular from ductal carcinomas, even in those infiltrating and in situ tumors with indeterminate features.^40^,^41^ Consistent with these findings, E-cadherin gene (CDH1) has been reported to be frequently mutated in infiltrating lobular carcinoma^42^–^44^ and lobular carcinoma in situ,^45^ but not in ductal tumors.^46^

An incidence of 4.1% of male breast cancer indicates that this disease is not that uncommon as presumed in this part of the world. The population of patients with various risk factors must be evaluated for such lesions. Breast cancer in men seems more frequently to be hormone-receptor positive and the *BRCA2* mutation confers a significant risk to men. Areas for future investigations are many and larger studies of pathological markers would be helpful to define which genetic abnormalities play a role in breast cancer in men and to determine which markers are important prognostic factors. It is further stressed that major epidemiological studies in populations at higher risk are needed to explore the various etiological factors.
